# Graft-versus-Leukemia Effect Following Hematopoietic Stem Cell Transplantation for Leukemia

**DOI:** 10.3389/fimmu.2017.00496

**Published:** 2017-06-07

**Authors:** Anne M. Dickinson, Jean Norden, Shuang Li, Ilona Hromadnikova, Christoph Schmid, Helga Schmetzer, Hans Jochem-Kolb

**Affiliations:** ^1^Haematological Sciences, Institute of Cellular Medicine, Newcastle University, Newcastle upon Tyne, UK; ^2^Third Faculty of Medicine, Department of Molecular Biology and Cell Pathology, Charles University, Prague, Czechia; ^3^Department for Hematopoietic Cell Transplantation, University Hospital Augsburg, Munich, Germany; ^4^Department for Hematopoietic Cell Transplantation, Internal Medicine III, Hospital of the University of Munich, Munich, Germany; ^5^Department of Hematology-Oncology Immunology Infectious Diseases, Klinikum München-Schwabing, Munich, Germany

**Keywords:** graft-versus-leukemia effect, animal models, donor lymphocyte infusion, allogenic natural killer cells, leukaemia associated antigens (LAA), LAA specific T cells, leukaemia derived dendritic cells, invariant natural killer T cells (i)NKT, leukaemia specific antigens

## Abstract

The success of hematopoietic stem cell transplantation (HSCT) lies with the ability of the engrafting immune system to remove residual leukemia cells *via* a graft-versus-leukemia effect (GvL), caused either spontaneously post-HSCT or *via* donor lymphocyte infusion. GvL effects can also be initiated by allogenic mismatched natural killer cells, antigen-specific T cells, and activated dendritic cells of leukemic origin. The history and further application of this GvL effect and the main mechanisms will be discussed and reviewed in this chapter.

## Introduction

Allogeneic hematopoietic stem cell transplantation (HSCT) transplantation has a major role in the treatment of leukemia and hematological disease, often the only treatment providing a chance of cure in otherwise refractory diseases. The primary approaches involved total body irradiation (TBI) (1) or cyclophosphamide (CY) (2). However, TBI alone was not immunosuppressive enough, and only one patient survived as a chimera (3). CY was readily immunosuppressive, but did not affect leukemic stem cells (2). Recovery from aplastic anemia was even observed after CY and graft failure, indicating the weak effect of CY on hematopoietic stem cells (4). Sustained success was reported with the combination of TBI and CY and other chemotherapy (5), as well as with the combination of CY and busulfan (6). In the 1970s and 1980s, interest focused on the conditioning treatment and the prevention of graft-versus-host disease (GvHD).

The first suggestion of a graft-versus-leukemia (GvL) effect was in 1956, using a mouse transplantation model, where rejected leukemia cells appeared to be eliminated by incoming bone marrow when irradiation was delayed (7). This led to the concept that the donor marrow cells may be responsible for the eradication of the leukemia.

This observation was applied to the clinic nearly 10 years later by Mathé and team in 1965. Mathé coined the term “adoptive immunotherapy” for the treatment of leukemia with allogeneic bone marrow transplantation (8) and showed that leukemia was eliminated by the GvL. In this paper, they also describe the “secondary syndrome,” later to be described as GvHD. Interestingly, bone marrow from six family donors were used for the transplant and in order to decide which donor may be used posttransplant to enhance GvL, the patient received skin grafts from all six donors. Histocompatibility tests at that time (9, 10) showed that the only skin graft, which was not rejected was the one which was closest genetically to the donor. This donor was subsequently used to give incremental doses of leukocyte treatment posttransplant and gave rise to GvL, but also GvHD. The latter was controlled by the use of hydrocortisone. The patient remained in remission for 1-year posttransplant but died from a viral infection with no sign of relapsing disease.

A major step toward successful transplantation was identifying HLA-identical siblings as best donors and syngeneic twins for prevention of rejection and GvHD. These findings were derived from dog experiments where the correct littermate was chosen by first ensuring that the donor leukocytes were negative, both against the donor in mixed lymphocyte reactions and with cytotoxic antisera (11, 12).

In 1990, over 2,000 transplants had been performed and reported by the International Bone Marrow Transplant Registry (IBMTR). An analysis of the transplants by Horowitz et al. showed conclusively that a GvL effect was important to reduce relapse. An increased relapse rate was observed when the graft was T cell depleted to prevent GvHD. In addition, the data showed that grafts with or without T cells had a high incidence of relapse, indicating that the antileukemia effect could occur independent of GvHD (13).

Relapse of residual disease is a common cause of reduced survival following HSCT. This occurs in 20–70% of patients and is dependent on several factors including pretransplant disease status, cytogenetic subtypes [in acute myeloid leukemia (AML) and in acute lymphoid leukemia (ALL)], stem cell source, age of the patient and donor, and type of conditioning regimen (14, 15). In addition, relapse contributes to 40–45% of deaths following HLA-matched identical HSCT and 34% in unrelated donor HSCT (16). The use of reduced intensity conditioning regimens has also led to GvL effects, which have been most marked in chronic myeloid leukemia (CML) and are also detectable in myelodysplastic syndrome (MDS), AML, and ALL.

This review describes the history and advances made in treating and or preventing relapse following HSCT using donor lymphocyte infusion (DLI), allogenic mismatched natural killer (NK) cells, antigen-specific T cells, and activated dendritic cells (DCs) of leukemic origin.

## Clinical Results Using DLI for Relapse after Hematopoietic Cell Transplantation

Although adoptive transfer of lymphocytes immediately after transplantation was attempted to induce remission, severe GvHD ensued and was unsuccessful for reducing relapse in high risk acute leukemia (17).

Careful studies in dogs given T cell-depleted marrow from dog lymphocyte antigen-identical littermate donors had shown that donor lymphocytes could be transfused after more than 2 months posttransplant without the risk of GvHD (18); this effect was subsequently shown in both canine and murine transplant models (18–21). Moreover, donor DLI converted mixed chimerism into complete chimerism, transferred immunity, and improved immune reactivity to leukemic antigens. Further work demonstrated the potential role of tolerance in the lack of GvHD development.

It took pioneering work in the 1990s (22, 23) to show that, for CML transplant patients, subsequent separation of the transplant by 2 months from the DLI enabled remissions and prevented GvHD. These first studies in man for CML were carried out by Kolb and colleagues with subsequent remission in all three patients for over a decade (22). Two of the three patients, treated in this first report of DLI, are still in complete remission (CR1) of CML (22). One patient had a cytogenetic relapse 20 years after DLI. She was retreated with DLI from her brother and responded again. The GvL effect has been confirmed for CML in numerous studies worldwide (24, 25) and are frequently durable offering potential cure for the majority of CML patients (26–28). These results have been collected by the European group for Blood and Marrow Transplantation (EBMT) (29), the US (26), and Japanese transplant centers (30). The best results with 70–80% cytogenetic complete remissions were reported for CML in cytogenetic and hematological relapse, other important factors being the presence of chronic GvHD prior to DLI and the time of relapse post-HSCT (31). Donor chimerism was also necessary for a successful GvL in CML. For patients with AML or MDS, the response rate to DLI is much lower (20–40%) and is lower still in ALL (10–13%) with intermediate results (40–52%), compared with CML, being obtained in multiple myeloma (MM) (32). In AML, DLI efficacy is thought to be limited to a small sub group of patients, those with favorable cytogenetics, with a low-tumor burden at relapse or in hematological remission prior to DLI (33). In general, complete remissions were durable in CML and only in a minority of patients with acute leukemia and MM (34).

## The History of the Role of T Cells and NK Cells in the GvL Effect

As stated previously, the role of T cells was further identified by studies using data from the IBMTR (35). In addition, differences in relapse rates with higher doses of TBI and fractionated TBI versus single-dose TBI were only seen in patients given T-cell-depleted marrow, while in patients given T-replete marrow, no differences were observed (35). In 2004, Ballester et al. described a graft versus myeloma effect after DLI and an autologous stem cell transplant rescue (36). Daguindau et al. described an antitumor effect of HSCT in 14 patients with either acute leukemia or MDS who sustained a long-lasting CR1, despite only transient or absent engraftment with donor cells (37). This effect, therefore, being caused by transient exposure to allogenic T cells and autologous reconstitution.

**Figure 1:**
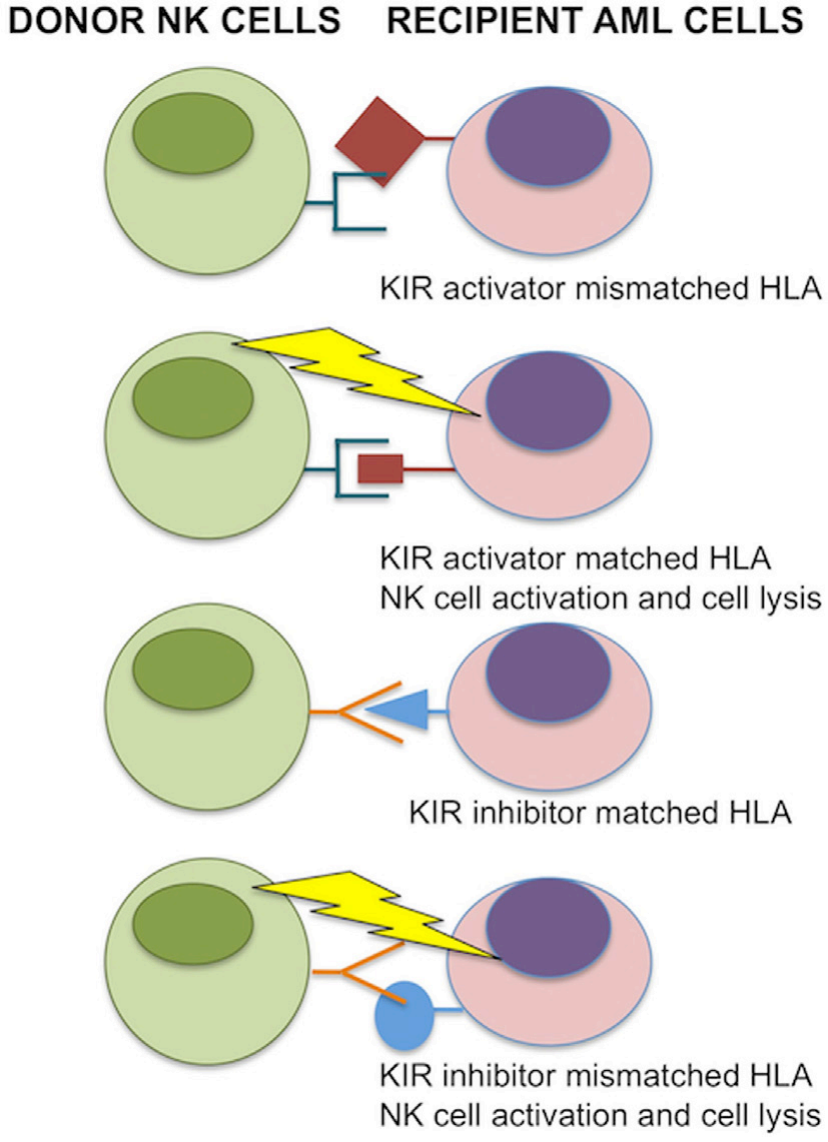
**Donor-versus-recipient natural killer (NK) alloreactivity**. NK cell function is regulated by KIR interactions with matched HLA class I alleles. If HLA is mismatched in transplant recipient leukemic cells, NK cells are relieved from inhibition and induce cell lysis. In the case for inhibitor KIRs, binding with matching HLA prevents donor NK cell activation to self. For activating KIRs, donor NK cells that bind the matched HLA are activated and induce cell lysis of transplant recipient acute myeloid leukemia (AML) cells. Image adapted from Ref. (48, 49).

**Figure 2:**
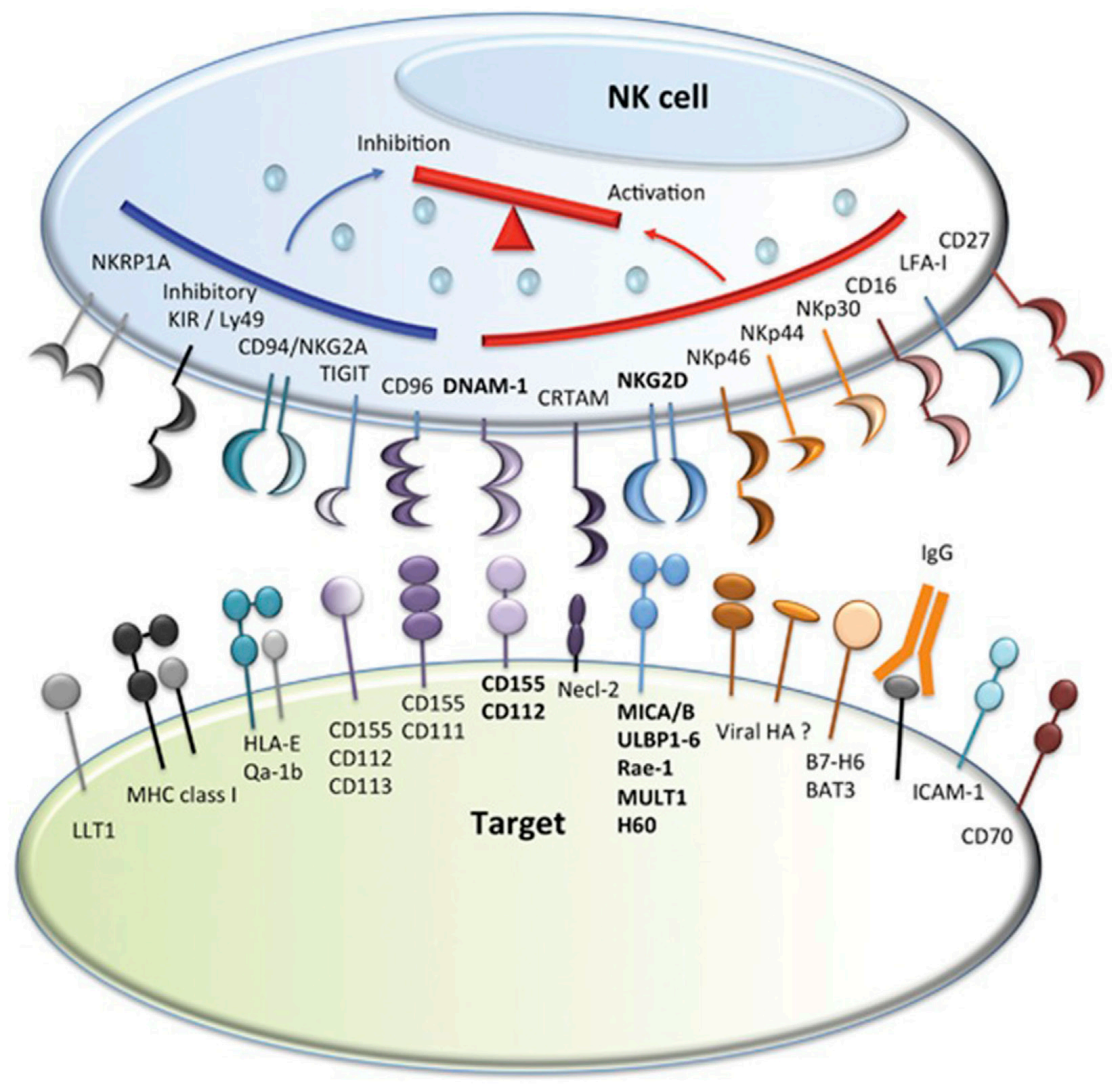
**Natural killer (NK) cell inhibitory and activatory receptors and their ligands**. Major inhibitory and activating receptors on NK cells and their cognate ligands on target cells. Image adapted from Ref. (46).

Natural killer cells were identified in the 1970s by Kiessling and Wigzell (38) and were shown to kill tumor cell lines in the absence of MHC class I molecules (39). This gave rise to Ljunggren and Karre in 1990 introducing the “missing self” hypothesis (40) where NK cells kill targets because they do not express high levels of “self” MHC class I gene products. They predicted the presence of receptors for self-MHC which, when engaged, would inhibit cytotoxicity. It was subsequently shown that NK cells can distinguish between normal and malignantly transformed cells by the presence of killer-cell immunoglobulin-like receptors (KIRs); these are receptors for MHC class I and are the main inhibitory receptors. The KIR inhibitory receptor family recognizes HLA-A, B, and C molecules (41, 42). Another inhibitory receptor C type lectin NKG2A recognizes the MHC molecule HLA-E (43–45). The inhibitory receptors give rise to a repertoire of NK cells with overlapping specificities. The function of NK cells is regulated by the balance of activatory and inhibitory signals transmitted by different cell surface receptors, such as KIRs, NK Group 2 member D (NKG2D), NKG2A/CD94, NKp46, and others (46, 47) (Figures 1 and 2). NK cells recognize both foreign and self-antigens expressed by NK-susceptible targets. NK cells attack cells lacking MHC class I molecules specific to the inhibitory receptors KIRs on the NK cells (47).

The biological and clinical effects of NK cells in allogeneic transplant have recently been reviewed by Benjamin et al. (50).

The first study utilizing NK cell alloreactivity was shown in 2002 by the group of Velardi (48). HLA haploidentical—mismatched family donors were used to transplant 57 AML patients and 35 ALL patients. Donor versus recipient NK cell reactivity was analyzed in groups with and without KIR ligand incompatibility in the graft-versus-host (GvH) direction. Protection from GvHD and AML relapse was observed and showed that KIR ligand incompatibility in the GvH direction predicted survival in AML patients. In contrast, in the ALL patients, KIR ligand incompatibility in the GvH direction had no effect on ALL survival rates. In this study, the transplant regimen was myeloablative and involved T cell-depleted grafts and very large doses of CD34 positive cells/kg, contributing to lack of GvHD and successful engraftment.

In a later study using non-myeloablative conditioning (2 Gy of TBI with or without fludarabine) and HLA-matched grafts, the risk of relapse was less in patients with ligands for all donor KIR, but this did not reach significance (51).

## Prophylactic and Preemptive DLI

Animal experiments were designed to demonstrate repletion of T cells by DLI after T-cell-depleted transplantation. In dogs, donor lymphocytes eliminated residual host hematopoiesis and converted mixed chimerism into complete chimerism (52). Following these results, prophylactic DLI became part of the FLAMSA regimen (fludarabine, cytarabine, amsocrine) for high risk AML (53). This regimen was designed for sequential therapy of high risk AML with FLAMSA followed by reduced intensity conditioning consisting of reduced TBI (4 Gy) or busulphan, antithymocyte globulin, and CY. DLI after immunosuppressive therapy was stopped for 30 days without development of GvHD. A matched pair analysis of patients receiving or not receiving DLI showed a significant advantage of patients given DLI; matching criteria were CR1 at day 120 from transplantation, absence of GvHD, and infection (54). Prophylactic DLI produced 80% long-term survival in several studies (55–58), involving around 340 AML and ALL patients. GvHD was seen in 28% of patients given DLI, but it was fatal only in 9% of all patients treated (58). However, in a small study of 12 patients in 2001, given DLI prophylactically as early as days 30, 60, and 90 days posttransplant, three patients developed GvHD (59). In a study of 15 patients treated with alemtuzumab in the conditioning treatment, seven patients developed GvHD and, in three patients, it was fatal (60). In lymphoma patients, conditioned with a regimen containing alemtuzumab for *in vivo* T-cell depletion followed by DLI for mixed chimerism, non-fatal GvHD occurred in 4 out of 17 patients (61). In our own unpublished evaluation, using landmark analysis on day 180, remissions were sustained in patients transplanted in CR. Relapse-free survival was improved in patients transplanted in an active phase of the disease. The median time of DLI posttransplantation was 160 days. Patients with active GvHD or relapse prior to 180 days were excluded from the evaluation. Fatal GvHD was observed in a patient treated for increasing mixed chimerism following an infection with Noro virus and another patient with upper respiratory tract infection. Viral infections can induce HLA class II antigens on non-hematopoietic cells leading to GvHD (62). Prophylactic antibiotic and virostatic treatment has been used to improve outcome.

Besides DLI for mixed chimerism, preemptive DLI can be given to patients with minimal residual disease (MRD) after transplantation. In CML, cytogenetic or molecular relapses indicate presence of residual disease without clinical signs; DLI have been effective in these patients with responses of >80% (16, 19, 47). In AML, there are a few molecular markers with sufficient sensitivity for diagnosing MRD. Monitoring WT1 gene transcripts has been found to predict relapse and the response to DLI (63) and RUNX1-RUNX1T1 transcript levels in patients with t(8;21) AML (64) pre DLI has been found to be predictive of a higher relapse incidence. MRD in acute leukemia in children and adults has been well documented (65) using a combination of flow cytometry and polymerase chain reaction, the latter for the detection of leukemia-specific fusion transcripts or clone-specific immunoglobulin including T cell receptor genes. In relapsed acute leukemia, a combination of gene transcript levels and four color flow cytometry, MRD monitoring has been found to predict a second relapse post-DLI (66). In myeloma, several groups have studied prophylactic or preemptive DLI (67–69), the rate of durable remissions is low, but secondary treatment is efficacious and survival is excellent.

The successful use of CML in DLI in the 1990s has been substantially reduced due to the reduced number of allo-HSCT for CML, to approximately 1% (70), by the success of tyrosine kinase inhibitors (TKIs) to treat CML. The family of TKI’s is capable of restoring complete molecular remission after relapse (71–73). CML relapse, molecular cytogenetic, or hematological has been reported as ranging from 16, 30, and 54%, respectively, using data from the Chronic Malignancies Working Party for the EBMT and based on 500 HSCT transplants from 1968 to 2004. The use of DLI in these cases was most successful if pre DLI factors such as chronic GvHD, cell dose, patient and donor gender mismatch, as previously described was taken into account (31).

In contrast, relapse after allo-HSCT for the other types of leukemia is further dependent in AML, on the age of the patients, disease status pre allo-HSCT, the AML sub types (primary or secondary), cytogenetic and molecular markers, type of conditioning and stem cell source (74–79). AML patients relapsing after allo-HSCT rarely responded to DLI although remissions have occurred in selected cases (26).

Use of DLI in a large cohort of 399 AML patients, collated from the Acute Leukemia Working Party of the EBMT, was associated with 21% overall patient survival at 2 years, compared with 9% for patients not receiving DLI (33). Better outcome was associated with lower tumor burden at relapse, female gender, favorable cytogenetics, and with patients in hematological remission before DLI or at the time of DLI. From these studies, an algorithm for the clinical use of DLI was developed for use in the treatment of relapsed AML, which included the sequence of cyto reductive chemotherapy or indication of CR1 prior to DLI (80).

Relapse after ALL varies from 30 to 35% depending on whether the patients have undergone a HLA-matched sibling transplant or matched unrelated donor (MUD) transplant (81), and response to DLI has been recorded at 50% with survival rates improved in patients who developed acute GvHD after DLI (82).

## Complications of DLI

### Graft-versus-Host Disease

Early experiments in canine, rat, and mice transplant models demonstrated no GvHD following infusion of non-sensitized donor lymphocytes into stable chimerisms (18–21).

This observation led to the concept that DLIs may be used to improve engraftment and accelerate immune reactivity without the occurrence of GvHD in a stable human chimera.

Contrary to the results in animal experiments with dogs and mice, GvHD was seen in humans given DLI (83). There are a great number of differences that may account for this. Unlike human patients, animals used for experiments are of younger age and are kept in protected environments, minimizing chronic infections and immune cross reactivity. More importantly, differences exist in the underlying malignant disease and its impact on alloimmunity as well as prior chemotherapy, depleting lymphocytes and ablating regulatory T cells (84).

Attempts at preventing GvHD included depleting cytotoxic CD8-positive T cells from the transfusion (85), arming T cells with suicide genes (86), and the administration of escalating doses (87), which was widely adopted for the treatment of recurrent CML (88).

An important role in the generation of GvHD after DLI is played by viral infections and/or reactivations of viruses (62, 89). Antiviral and antimicrobial prophylaxis has, therefore, prevented viral infections and improved the response to DLI.

In addition, studies by the Chronic Malignancies Working Party of the EBMT (29, 83, 90) have shown that up to 40% of patients with secondary GvHD post-DLI had a twofold to threefold increased risk of death, compared to patients without GvHD. The best results following DLI are obtained when patients obtain remission without GvHD, thus separating GvL from GvHD in these patients (90–92).

A recent study by Radujkovic et al. aimed to identify pre-DLI factors, which may predict survival in remission without secondary GvHD in patients with relapsing CML. The study identified that the presence of chronic GvHD before DLI and less than 1 year between the allo-HSCT and DLI were associated with inferior survival. The likelihood of survival in remission without GvHD was most prevalent, i.e., 50% at 5 years follow-up, when DLI was given without prior chronic GvHD and greater than 1-year post allo-HSCT for molecular and/or cytogenetic CML relapse.

For hematological relapse, a T cell dose of greater than 50 × 10^6^/kg, the donor–recipient gender mismatch and prior chronic GvHD were the worst prognostic factors.

Initial studies showed that starting the transfusion of donor cells at low cell numbers followed by escalating doses until response or induction of GvHD reduces the incidence and severity of GvHD but preserves a GvL effect (87, 93).

Guglielmi et al. subsequently analyzed 344 CML patients treated by DLI and their study found that the initial cell dose was given based on donor type (HLA-identical sibling or HLA matched volunteer unrelated donor), T cell depletion, GvHD prior to relapse, relapse type (cytogenetic, molecular, and hematological), and year (90). The lower the initial cell dose, the high number of subsequent transfusions were given to achieve a response and the incidence and severity of GvHD and myelosuppression increased with the higher initial cell dose of greater than 0.2 × 10^6^ MNC/kg. Factors such as donor type, gender of donor, disease phase at transplantation, T cell depletion, interval from transplantation to DLI, GvHD prior to relapse and relapse type, all influence outcome post-DLI and potential incidence of GvHD and prolonged survival.

### Myelosuppression

Pancytopenia and marrow aplasia have been observed in patients treated with DLI for hematological relapse of CML (92) and transfusion of marrow from the original donors restored hematopoiesis (94). Sometimes, myelosuppression and marrow aplasia were sudden and related to acute GvHD (95). This type of phenomenon can be explained by the incoming donor marrow removing leukemic hematopoiesis prior to donor hematopoiesis being fully established. Lack of recovery in some patients may also be explained by too few stem cells in the donor graft to sustain hematopoiesis.

## Improvement of the Response

Complete cytogenetic responses were achieved initially in patients with CML who had been treated with massive doses of donor lymphocytes (>10^8^/kg) and interferon-α (IFN-α) but had failed to respond to IFN-α alone. Responses were better in patients treated with myeloablative conditioning and T cell-depleted grafts than in patients with non-myeloablative conditioning and peripheral blood (PB) stem cells containing high proportions of T cells (29, 96). The better response of myeloid forms of leukemia led to the hypothesis that direct antigen presentation by leukemia-derived DC may play a major role in the activation of donor T cells (91). IFN-α improves the GvL effect of low doses of DLI and the combination of IFN-α and granulocyte macrophage-colony stimulating factor (GM-CSF) improved antigen presentation and generation of cytotoxic T cells in CML (97). Consequently, patients not responding to IFN-α and DLI responded to the combination of DLI, IFN-α, and GM-CSF. Moreover, T cells of CML patients displayed reduced zeta-chain expression and tended to go into apoptosis, which could be reversed by IFN-α (98). Lymphodepletion prior to DLI may enhance the anti tumor effect of the infused T cells, however, this can cause more GvHD (84).

Future potential improvements may come from the treatment with checkpoint inhibitors in order to increase T cell activation by inhibiting downregulation (99). Preliminary reports have shown feasibility of single doses of ipilimumab with blockade of CTLA4, without producing GvHD (100). An attractive way of treatment may be the use of central memory T cells that maintain a memory immune reaction without producing GvHD (101).

### Antigen-Specific T Cells

In acute leukemia, the pace of the disease is too fast to allow the development of immune reactions against the leukemia as observed for CML. Therefore, the generation of specific tumor immune T cells for the rapid elimination of leukemia cells have been investigated, and there are several candidate antigens, which have been used in assessing immune reactions to leukemia and induction of remission.

### Leukemia-Associated Antigens (LAAs)

Leukemia-associated antigens are often overexpressed in leukemia blasts and absent in normal tissue such as Wilms tumor 1 (WT-1), preferentially expressed antigen in melanoma (PRAME), melanoma family antigen (MAGE), receptor for hyaluron mediated motility (RHAMM), testis antigens like New York esophageal squamous cell carcinoma-1 cancer-testis antigen (NY3ESO), and granulocyte antigens such as PR1 (a 9 amino acid HLA-A*0201-restricted peptide derived from proteinase3).

The most widely studied antigen is coded by WT-1, a gene involved in Wilms tumor and present in about 77% of AML (102). Cytotoxic T cells against WT-1 kill AML stem cells preventing engraftment in NOD/SCID mice (103). Most immune reactivities found after DLI against any of these antigens are weak. Moreover, immune reactivity against autologous antigens is frequently suppressed by mechanisms of tolerance mediated by, e.g., regulatory T-cells (Tregs) or inhibitory cytokines (104). Peptides presented by foreign HLA antigens can be immunogenic by different configurations (105). It has been shown in a mouse model that T cell receptors cloned from HLA-different T cells can be transduced into autologous T cells to maintain immunity (106). Mispairing of T cell receptor chains with endogenous TCR chains could be avoided by lentiviral (LV) transfer and more recently by silencing endogenous TCR with endonucleases prior to transduction (107). Our own results correlated stable remission after HSCT with the presence of higher proportions of LAA-specific T-cells. The simultaneous detection of two different LAA-specific (CD8-positive T-cells) correlated with a higher chance of long-lasting remission. Moreover, we detected clonally restricted (PRAME-)specific T-cells and, in general, an enrichment of (effector)memory T-cells in cases with stable remission (102).

Our studies focused on patients after HSCT and might, therefore, be in accordance with the finding of PRAME reactive cytotoxic T precursor cells in healthy donors and not in AML patients (108). Encouraging results for PRAME as a target for immunotherapy in leukemia were, however, reported by Rezvani and colleagues (109). Immune response to RHAMM has also been elicited by vaccination (110). At present, lasting success using anti-LAA T cells have not been reported.

### Leukemia-Specific Antigens

Leukemia-specific antigens are antigens coded for by a mutational event in the leukemic clone. A unique translocation is the cause of CML, and the peptides derived from this gene fusion are presented by HLA antigens, the most immunogenic by HLA A3. In addition, DC in CML patients have the BCR/ABL translocation and can stimulate allogeneic T cells, inducing a cytotoxic T cell effect (97).

Single cases of enduring responses and immunity to the fusion peptide have been reported with peptide vaccine and interferon-α (104). In AML, many different mutations make the production of a vaccine to leukemia-specific antigens difficult, but cytotoxic T cells against nucleophosmin (NPM1) have been reported (111). NPM1 may be a preferable target, because of its presence on leukemia stem cells. New possibilities may arise from the detection of immune inhibitors of T cell activity, which may be reconstituted by check point inhibitors enabling T cells to react to whatever antigen is recognized. In AML and myeloid malignancies, other mechanisms (33) of T cell suppression may also be important. For example, blockade of CD47 expression on tumor cells, driving macrophage T cell and dendritic cell activation leads to tumor cell killing (33).

### Minor Histocompatibility Antigens (mHAs)

Minor histocompatibility antigens are responsible for graft rejection and GvHD in HLA-identical sibling transplants, they may be effective in GvL, if these antigens are expressed on hematopoietic cells. The reaction against hematopoietic cells of the patient is irrelevant, because hematopoiesis is substituted by donor cells. Both CD4-positive and CD8-positive T cells respond to leukemic or minor histocompatibility antigens. In the latter case, cytotoxic T cells have been generated against CML, which have induced remission and shown to be correlated with the presence of cytotoxic T lymphocytes against minor histocompatibility antigens HA1 and HA2 (112). In an EBMT study, responses of patients with recurrent CML were only seen in those with an allogeneic donor, syngeneic twin donors did not respond (29). There are mHAs with a tissue distribution restricted to the hematopoietic system (113) and mHA expressed in all tissues. The strongest mHAs are those coded by the Y chromosome (114). Y chromosome antigens-directed T cell responses show strong antileukemic effects (115), but they may also produce GvHD. mHA also produce peptides presented by both HLA class I and HLA class II, as well as eliciting antibodies against themselves (116). We have investigated Y chromosome genes by comparing leukemic blasts with normal monocytes, and we found four genes overexpressed in AML (102). Peptides of the gene products were loaded onto T2 cells and cytotoxic T cells could be produced against two of the four antigens. As demonstrated in a canine model, we could demonstrate that female dog-effector-T-cells could be specifically stimulated against male chromosomal (UTY−) gene products (116).

The search for new mHA was expanded to study the reactivity of T cells against antigens and overlapping peptides of genotyped lymphoblastoid cell lines (HapMap-Project) (117). Several new antigens were found, but the majority of reactions did not show significance probably due to additional factors provided by the microenvironment (118).

### Production of DC of Leukemia Origin (DC_leu_)

**Figure 3:**
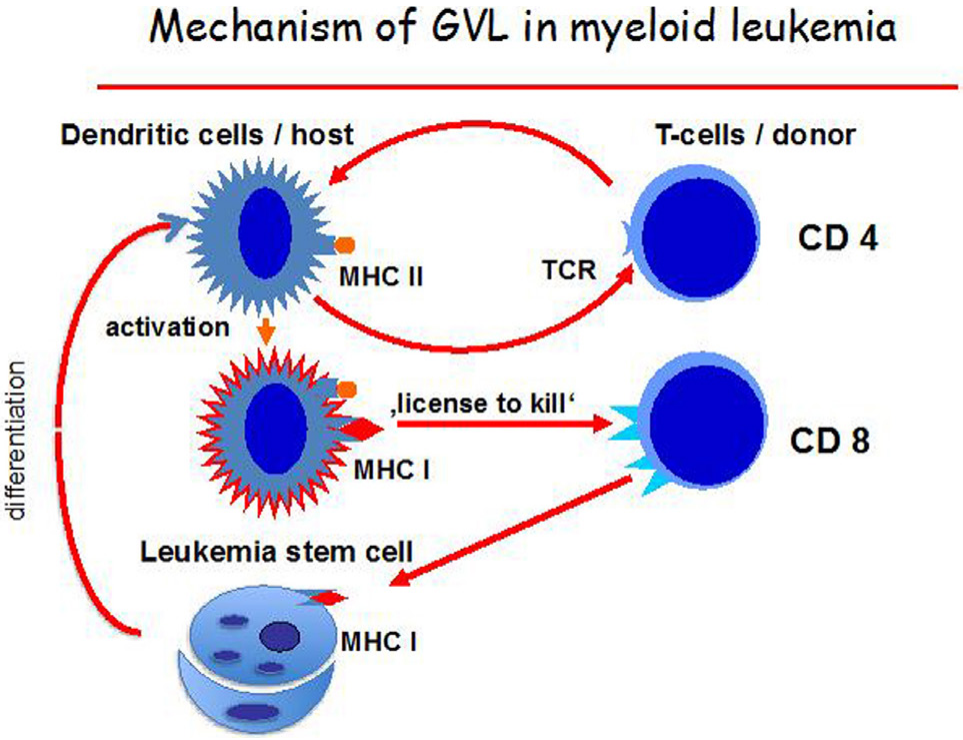
**Mechanisms of the graft-versus-leukemia (GvL) effect**. Host dendritic cells (DCs), activated by CD4-positive T cells, mature and can then activate CD8-positive T cells *via* a “license to kill” and attack leukemia cells. The leukemia cells themselves can also differentiate into self DC.

Effective antigen presentation is essential in GvL reactions. GM-CSF has been effective in the production of DC of leukemia origin (DC_leu_), which were able to induce cytotoxic T cells (119). Inhibition of cytotoxicity was greater with antibody against HLA class I than antibody against HLA class II. Production of DC from AML leukemia blasts was extensively studied by Schmetzer and colleagues (102, 120) (Figure 3). It could be shown that DC_leu_ could be successfully generated from blood samples in every AML patient using methods of DC generation containing different mixtures of immune-modulatory factors, GM-CSF, IL-4, TNF-α, FLT3-L, IL-1β, IL-6, PGE2, bacterial lysate of streptococcus pyogenes (PICIBANIL), or calcium ionophore.

Moreover, Schmetzer’s group correlated a successful *ex vivo* generation of DC_leu_ from AML blasts before HSCT with a clinical response to HSCT or DLI (102), which may suggest a central role of DC_leu_ in priming antileukemic T cells.

*In vivo* Schmetzer’s group has shown that rats heavily diseased with promyelocyte-like leukemia and treated with DC_leu_-inducing Kits GM-CSF with Picibanil, prostaglandin 1 or 2 (PGE1 or PGE2) (patent-number 10 2014 014 993) showed a highly significant reduction of blasts, an increase of memory like T-cells, and a decrease of Treg after two Kit-applications in only 9 days, therefore, suggesting that as proof of principle, a DC_leu_ induction *in vivo* could lead to T-cell activation resulting in a specific blast reduction. Our ongoing research focuses on the transfer of this strategy to patients with AML (121) [Christoph Schmid and Helga Schmetzer, personal communication; (102, 122)].

Some patients with AML relapsing after transplantation could be induced into remission by treatment with low-dose cytarabine, mobilized donor cells including PB stem cells and post-grafting GM-CSF (123). GvHD occurred on the day after T cells were administered. The remission rate was doubled compared to the results of an EBMT study and some patients survived in long-term remission without further treatment. Risk (33) factors for failing treatment were early relapse (less than 180 days) and failure to respond to low-dose cytarabine. In the EBMT study, similar risk factors were defined and long-term survival was only seen in patients treated with DLI or second transplants after achieving CR1 (80). Several centers have shown efficacy of repeated treatments with azacitidine and DLI also establishing long-term remission in some patients (124). Azacitidine and cytarabine have the potential to induce differentiation of blasts, new targeted drugs like sorafenib (125) and midostaurin (126) may also be helpful in Fms-related tyrosine kinase 3 (FLT-3)-positive leukemia or panobinostat, an oral deacetylase inhibitor for MM (127, 128).

Both cytokines and targeted drugs enable leukemia blasts to differentiate into DC which, by presenting antigen, provide stimulating signals to donor naïve T cells (129). Both reactive CD8-positive and CD4-positive T cells have been found in patients responding to DLI (130). *In vitro* generated T cell cytotoxicity predominantly consisted of CD8-positive T cells, but CD4-positive cytotoxic T cells were also present (119). Schmetzer et al. have shown that T-cell clonality was more restricted after *ex vivo* DC_leu_ induction compared to blast stimulation, pointing to a role of DC_leu_ to efficiently enrich selected T-cell-clones. Interestingly, T cells with the same Vβ chain of the T cell receptor that was observed *in vivo* were also found in *in vitro* cultures (131).

The production of CD4-positive T cells against HLA-class II restricted minor antigens have the advantage that HLA-class II antigens are only expressed on cells of the hematopoietic system. The GvH reaction of allogeneic T cells, therefore, spares non-hematopoietic organs and is operationally leukemia specific (132). However, there are limitations to this approach, since inflammation of healthy tissue increases the expression of HLA-class II on non-hematopoietic cells and induces GvHD. Like normal hematopoietic stem cells, leukemia stem cells are quiescent and do not express HLA-class II antigens (133). Therefore, HLA-class I restricted CD8-positive T cells may be further required for complete elimination of leukemia stem cells.

The hypothetical mechanism behind the GvL effect is that DCs become activated by donor CD4-positive T cells and, once mature, the DCs activate CD8 positive T cells by a mechanism called “license to kill” (134). These also react against leukemia stem cells until the pool of these cells is depleted. CD8 positive naïve T cells, therefore, become involved in the GvL reaction with antigens involving HLA class II and class I peptides being the optimal target (Figure 3). Presumably, repeat interactions of host/leukemia-derived DCs and donor CD4-positive T cells are necessary for sustained GvL effects. Direct antigen presentation by host/leukemia DCs may also be a further mechanism.

Another possible way the GvL reaction is maintained is by central memory T cells that do not require CD4 help for sustained GvL effects. Precursor cells recognizing LAA and mHA are found in low frequencies in the bone marrow of healthy donors, and these can be expanded by encountering some of these antigens in the patient. Examples of the efficacy of memory T cells are virus-specific T cells that can be selected from immune stem cell donors and transferred to the patient (135). As these cells can expand *in vivo*, the presence of central memory T cells recognizing antigens on leukemia cells in the graft is the most important criteria for successful GvL effects. *Ex vivo* data of Schmetzer and colleagues showing increased memory T cell proportions after T cell stimulation with DC_leu_, compared to blasts, and reduced naïve T cells support this view (102).

## Mechanisms of a GvL Effect—NK Cells

T cells and NK cells in the donor graft eliminate residual leukemia cells in the patient by T cell interaction with leukemia-specific antigens or mHA, activated NK cells interact with allogeneic targets lacking killer immune receptors (48, 136) (Figure 2).

Natural killer cells are the major players of innate immunity with the fastest reconstitution *in vivo*. NK cells are the earliest lymphocytes recovering after HSCT and due to delayed reconstitution of a functional T cells repertoire, NK cells are a vital lymphocyte subset exerting antileukemic effects and have been linked to reduction in relapse rates or improved disease-free survival (137). Nevertheless, as recently reviewed by Zhao et al., the reconstitution of NK cells is influenced by many factors, including the conditioning regimen, level of T cell depletion, and the use of immune suppression after transplantation (138).

### KIR–Ligand Interactions and HSCT Outcome

Many clinical studies have linked NK cells to successful outcomes following HSCT. For instance, the donor KIR genotype plays an important role in the development of infections posttransplant. Recipients of unrelated donor HSCT from donors with an activating (KIR) (B/x) genotype have less infectious (bacterial) complications than those with an A/A KIR genotype, because of the enhanced NK cell function (139). It also has an effect on survival post HSCT; in a small study of HLA-matched T cell-replete sibling transplants, better overall survival was associated with the presence of group B KIR haplotypes in the recipient and the absence in the donor (140). Conversely, three donor B haplotype KIR genes have been reported to be associated with reduced relapse and improved overall survival in a study of HLA-matched T cell-replete sibling transplants (141), and in a cohort transplanted for AML, donor possession of group B KIR haplotypes was associated with improved relapse-free survival but a higher incidence of chronic GvHD (142). The group B KIR haplotype KIR3DS1 in the donor has been found to be associated with decreased acute GvHD in MUD transplantation; however, this effect appears to be unique to this specific B allele (142).

Moreover, in haploidentical HSCT, NK cells may express inhibitory killer immunoglobulin-like receptors that are not engaged by any of the HLA class I alleles present on recipient cells. Such “alloreactive” NK cells greatly contribute both to eradication of leukemia blasts escaping the preparative regimen and to clearance of residual host DCs and T lymphocytes (thus preventing GvHD and graft rejection, respectively) (143).

Furthermore, in umbilical cord blood (UCB) transplants for acute leukemia in first CR1, patients with KIR ligand incompatible donors had improved overall survival (57 versus 40%) and decreased relapse (20 versus 37%) when compared with those without these incompatibilities. Benefits of KIR ligand incompatibility were most striking among patients with AML although UCB recipients with ALL also had a trend toward improved leukemia-free survival (144).

IPH2101 is a human IgG4 monoclonal antibody directed against inhibitory KIRs (KIR2DL-1, -2, and -3), which blocks KIR–ligand interaction and augments NK cell-mediated lysis of HLA-C-expressing tumor cells. A phase I trial of IPH2101 (NCT00552396) was conducted in 32 patients with relapsed/refractory MM suggesting that IPH2101 is safe and tolerable at doses that achieve full inhibitory KIR saturation (145).

### *In Vitro* and *In Vivo* Induction of a GvL Effect

Lentiviral vectors have been successfully used to transduce both T and NK-cell lines. Chimeric antigen receptors (CARs) are synthetic engineered receptors that target surface molecules in their native conformation, independent of MHC and of antigen processing by the target cells (146). For example, CS1 is a surface protein highly expressed on MM cells and is amenable to targeting with CS1-specific CARs. CS1-CAR-transduced NK cells showed stronger cytotoxic activity against CS1-expressing MM cells and showed increased IFN-γ production compared with mock-transduced NK cells. In an orthotopic MM xenograft model, adoptively transferred CS1-CAR-NK-92 cells suppressed the growth of human IM9 myeloma cells and significantly prolonged mouse survival (147). Moreover, CAR-NK cells may be safer compared with that of CAR-T cells, because of lack of *in vivo* clonal expansion and cytokine storm.

Novel research techniques use either all or part of an antibody structure to deliver enhanced effector activity to the tumor site. Bi-specific killer engagers (BiKEs) are constructed with a single-chain Fv against CD16 and a single-chain Fv against a tumor-associated antigen. The mechanisms by which BiKEs potentiate NK effector functions include intracellular calcium mobilization through direct CD16 signaling (148). Fully humanized CD16 × CD33 BiKEs have been shown to trigger NK-cell activation *in vitro* against CD33+ AML cell lines and primary refractory CD33+ AML targets (149). BiKEs enhance degranulation and cytokine production by NK cells derived from patients with MDS and cultured with CD33+ AML cell lines, irrespective of disease stage and age stratum (150). A potential drawback of this approach is the relatively short half-life of the antibody constructs, with limited trafficking to the tumor site.

### *Ex Vivo* Expansion of NK Cells and Induction of GvL Effects

There is no indication to suggest that human GvHD is linked with NK cell infusions, thus increasing the NK cell dose is one useful approach to improve the antileukemia activity. However, for clinical therapy one significant limitation is that the numbers of NK cells/kg recipient weight obtained by leukophoresisis are relatively small (~2 × 10^7^/kg).

Classically, GMP-compliant NK-cell products have been generated from peripheral blood mononuclear cells collected by apheresis (151). IL-15 was used to promote NK-cell proliferation and survival and has been variably used in GMP-grade laboratory protocols. Different expansion methods rely on human feeder cells including artificial antigen-presenting cells that are modified with costimulatory molecules, such as CD137 ligand and membrane-bound (mb) IL-15 or IL-21. However, expanded NK cells may affect the replicative potential and long-term viability of *in vivo* infusion. For instance, in NK cells, both FAS expression and susceptibility to apoptosis are increased after co-culture with IL-2 or with feeder cells (152). In addition, some receptors required for homing were reduced in expanded NK cells, such as CCR7, a member of the G protein-coupled receptor family. In line with this, NK cells expanded with genetically modified K562 cells were shown to predominantly express a CD16^+^CD56^+^ phenotype, with no detectable CCR7 (153, 154). To obviate this, NK cells have been co-cultured with genetically modified, IL-21/CCR7-expressing K562 cells, in order to transfer CCR7 onto NK cells *via* trogocytosis. CCR7 expression occurred in 80% of expanded NK cells within 1 h (154).

Umbilical cord blood is an emerging source of NK cells for clinical applications and also provides an *in vitro* system to analyze NK development (155). However, NK cells from PB and UCB differentially express cytokine receptors, for instance, IL-15Rα being preferentially detected on UCB NK cells and IL-12Rβ1 and IL-18α receptors being primarily found on PB NK cells (156, 157). This implies that, unlike PB NK cells that are fully activated by IL-2 alone, UCB NK cells may require additional cytokine stimuli (158). For instance, the addition of tacrolimus and low-molecular-weight heparin significantly enhances NK-cell expansion induced by IL-2, IL-15, and anti-CD3 mAbs (159).

Like UCB, human embryonic stem cells (ESCs) as well as induced pluripotent stem cells (iPSCs) are also potential sources of phenotypically mature and functional NK cells. ESCs and iPSCs were first used to produce hematopoietic progenitors with the “spin embryonic body (EB)” method, in which defined numbers of cells were spin-aggregated in serum-free medium. Spin EB-derived cells were then tested in a feeder-free and serum-free system containing NK-cell promoting cytokines, i.e., IL-3, IL-7, IL-15, SCF, and Flt3-L. Importantly, NK cells developed in similar numbers, phenotype, and functional characteristics as those differentiated with the use of murine stromal cells (160).

Several malignant NK cell lines were established and used for clinical trials in some countries, as reviewed elsewhere (161). The adoptive transfer of NK cell lines has theoretical advantages related to lack of expression of inhibitory KIRs, lack of immunogenicity, and ease of expansion. For instance, K562-mb15-41BBL cells were used to expand NK cells transduced with an anti-CD19-BB-ζ CAR and showed enhanced reactivity to CD19+ leukemia cells (162). Similar to K562-mb15-41BBL, K562 genetically modified to express mbIL-21, or to co-express the ligand for 41BB and the NKG2D ligand MICA (K562-4-1BBL-mMICA), have been shown to promote large-scale expansion of NK cells with enhanced antitumor *in vitro* reactivity (163–165). Moreover, EBV-immortalized B-lymphoblastoid cells (EBV-BLCL) are known to strongly support NK cells *in vitro* expansion and antitumor activity (166–168). Escudier and colleagues used 35-Gy-irradiated LAZ 388 EBV-BLCL for the *ex vivo* expansion of NK cells from patients with metastatic renal cell adenocarcinoma. Based on their protocol, a phase I clinical trial is currently investigating technical feasibility and clinical efficacy of large-scale NK infusions (up to 1 × 10^9^/kg) in cancer patients receiving bortezomib administered with the scope of increasing susceptibility of tumor cells to NK-mediated lysis (169, 170).

In addition, Schmetzer and colleagues have shown recently that invariant (i)natural killer T ((i)NKT) and cytokine-induced killer (CIK) cells, where both cell types combine the characteristics of T as well as NK cells, and their subsets are promising cells in the induction of antileukemic reactions. Preliminary findings show that proportions and compositions of these cells provide prognostically relevant data for patients with AML, ALL, and CLL. Moreover Kit-treated AML-blasts (resulting in DC_leu_) induce a shift not only of T-cells but also of iNKT, NK, and CIK cells counts and proportions, correlating in improved antileukemic activity against AML blasts and implying cross talk between these cells (171).

## Treg Therapy and Its Effects in GvL and Relapse

The effects of Treg therapy for GvHD, GvL, and relapse post-HSCT has been recently summarized by Romano et al. (172). There have been several trials investigating the safety and efficacy of Treg-based therapy, the first recorded trial was reported in 2009 where *in vitro* expanded CD4+ CD25+ CD27− cells were used for the treatment of two patients with either acute GvHD or chronic GvHD (173). The patient with chronic GvHD showed a significant improvement of symptoms and the patient with acute GvHD had a transient improvement, however, due to the very low patient number no conclusions on the effect of Treg therapy on relapse were drawn. Another trial in 2011 reported the use of expanded Tregs from third party UCB in 23 patients with acute GvHD (174), no toxicities were documented and GvHD was reduced with no effect on relapse when compared with 108 historical controls.

Edinger and Hoffmann reported a small phase I safety trial (175) where nine patients at high risk of relapse post-HSCT were treated with freshly isolated donor Tregs, then 8 weeks later, conventional T cells were given to promote GvL activity and, as in the previous trial, there was no increased risk of relapse. In 2014, a group reported a trial in 43 patients with high risk leukemia, using freshly isolated donor Tregs pre-haploidentical HSCT, to avoid intensive *ex vivo* depletion of T cells in the graft. This protocol showed for the first time that adoptive immunotherapy with Tregs protected against GvHD compared to patients undergoing conventional haploidentical transplants. In addition, the incidence of relapse was reduced, suggesting that Tregs do not target GvL (176). More recently, a trial of the infusion of expanded Tregs and IL-2 for chronic GvHD has been reported. The study showed that the stability and functionality of the Tregs *in vivo* was maintained due to the increase in the number of T cells post-infusion. There was no toxicity nor exacerbation of chronic GvHD or other adverse immune reactions, chronic GvHD responded but the trial had only five patients and two of these patients developed non-hematological malignancies suggesting that Tregs may contribute to a tumor escape mechanism *via* suppression of the immune response (177).

## Conclusion

Many questions remain to be addressed in order to optimize the GvL effect of DLI for treating and preventing relapse and Table 1 summarizes the main features of this review. In CML, long-term remissions can readily be obtained by the treatment with low-dose IFN-a and DLI, in AML, long-term remissions may be obtained by a more aggressive approach involving mobilized stem cells and GM-CSF following cytarabine or repeated treatments with targeted drugs like azacytidine, sorfenib, midostaurin, immune-modulatory/blastmodulatory Kits, and DLI. Better results may be achieved with prophylactic or preemptive DLI and/or treatment with blast-modulating Kits combining myeloid cell-differentiating factor GM-CSF with “danger”-signaling and DC-maturation-inducing factors (e.g., PGE1, PGE2, or Picibanil) addressing myeloid blasts and converting them to DC_leu_—resulting in an *in vivo* stimulation of antileukemic T-cells. In addition to measuring minimal residual disease (MRD) or mixed chimerism, prophylaxis for viral infections may be required to avoid triggering of GvHD.

**Table 1:** **Overview of the chapter and summary of main points**.

Chapters	Sub headings	Reference	Main points
Introduction		(1–16)	Introducing hematopoietic stem cell transplantation (HSCT), graft-versus-leukemia (GvL), and residual disease
Clinical results using DLI for relapse after hematopoietic cell transplantation		(17–34)	First studies of donor lymphocyte infusion (DLI) for chronic myeloid leukemia (CML) and results for acute myeloid leukemia (AML) and acute lymphoid leukemia (ALL)
The history of the role of T cells and NK cells in the GvL effect		(35–48, 50, 51)	Early studies of the role of T cells and natural killer (NK) cells
Prophylactic and preemptive DLI		(52–81)	Studies in CML; AML and ALL
Complications of DLI	Graft-versus-host disease	(82–92)	Factors affecting GvHD occurrence and DLI
	Myelosuppression	(91, 93, 94)	Myelosuppression can be related to aGvHD
Improvement of the response	Antigen-specific T cells	(29, 90, 95–102)	Overview
	Leukemia-associated antigens (LAAs)	(101–109)	Overview of antigens expressed in leukemia blasts and absent on normal tissue
	Leukemia-specific antigens	(33, 96, 103, 110)	These are antigens coded for by the mutation event in the leukemic clone
	Minor histocompatibility antigens (mHAs)	(29, 111–117)	mHA, responsible for graft rejection and GvHD in HLA identical siblings
	Production of DC of leukemia origin (DC_leu_)	(80, 101, 118–133)	Cytokines and certain drugs cause leukemic blasts to differentiate into DC, which can then stimulate GvL
Mechanisms of a GvL effect—NK cells		(48, 136–138)	Activated donor NK cells induce GvL by interaction with allogeneic targets lacking killer immune receptors
	KIR–ligand interactions and HSCT outcome	(139–145)	Regulate the killing function of NK cells, most are inhibitory, they are pleomorphic and their genotype is important in GvL
	*In vitro* and *in vivo* induction of a GvL effect	(146–150)	Chimeric antigen receptors (CARs) T cells and CAR NK cells have shown promise and more recently Bi-specific killer engagers have been developed
	*Ex vivo* expansion of NK cells and induction of GvL effects	(151–170)	NK cells can be expanded from peripheral blood mononuclear cells, umbilical cord blood, ECSs, and also malignant NK cells lines but all have their limitations
Treg therapy and its effect in GvL and relapse		(172–177)	Tregs shown to reduce GvHD and do not induce relapse, but patients may potentially develop non-hematological malignancies

In myeloma, antigen presentation may be a problem and dendritic cell vaccines as well as low-dose lenalidomide may be helpful for sustained remissions. Antibodies-engaging effector cells are still to be studied in the allogeneic situation. Finally, the role of CD4-positive T cells and their interaction with CD8-positive T cells remains to be demonstrated on leukemia stem cells.

Clinical therapy with NK cells has been inspired by recognition of their potent antileukemia activity. The studies discussed above provide a solid basis for development of NK cell trials for leukemia therapy while minimizing risks (151). To advance NK cell therapies, both further study of basic NK biology (including iNKT and CIK cells) as well as a better understanding of interactions with other immune cells will be required (171). Unmanipulated bone marrow followed by CD6-depleted mobilized blood cells produced long-term remissions in advanced cases of acute leukemia; CD6-depleted PBSC provides NK cells, stem cells, and a minority of suppressive CD8-positive cells (178). Recently, excellent results have been reported in ALL and lymphoma patients with HLA-haploidentical transplants and high-dose cyclophosphamide after transplantation (179, 180). Even HLA haploidentical DLIs were possible in cases of relapse with excellent results in Hodgkin’s disease (181).

## Author Contributions

All authors contributed to the manuscript and AD, HJ-K, and HS reviewed the contents.

## Conflict of Interest Statement

The authors declare that the research was conducted in the absence of any commercial or financial relationships that could be construed as a potential conflict of interest.
